# Cluster analysis of heart rate variability reveals subgroups with preserved and early-impaired autonomic regulation in amyotrophic lateral sclerosis

**DOI:** 10.1186/s12883-026-04901-w

**Published:** 2026-05-01

**Authors:** Juri Sawada, Yuki Nakayama, Keiichi Shimatani, Chiharu Matsuda, Michiko Haraguchi, Yumi Itagaki, Kota Bokuda, Kentaro Hayashi, Ryo Morishima, Toshikazu Shinba, Sakiko Fukui, Toshio Shimizu

**Affiliations:** 1 Unit for Intractable Disease Nursing Care, Tokyo Metropolitan Institute of Medical Science, 2-1-6 Kamikitazawa,Setagaya-ku, Tokyo, Tokyo, 156-8506 Japan; 2 Department of Home Health and Palliative Care Nursing, Graduate School of Health Care Sciences, Institute of Science Tokyo, 1-5-45, Yushima, Bunkyo-ku, Tokyo, 113-8510 Japan; 3 Center for Preventive Medical Sciences, Chiba University, 1-8-1, Inohana, Chuo-ku, Chiba, Chiba-shi, 260-0856 Chiba Japan; 4 Department of Neurology, Tokyo Metropolitan Neurological Hospital, 2-6-1, Musashidai, Fuchu-shi, Tokyo, 183-0042 Japan; 5 Department of Psychiatry, Shizuoka Saiseikai General Hospital, 1-1-1,Ojika,Suruga-ku, Shizuoka-shi, Shizuoka, 422-8527 Japan; 6 Tokyo Metropolitan Fuchu Medical Center for the Disabled, 2-9-2, Musashidai, Fuchu-shi, Tokyo, 183-0042 Japan

**Keywords:** Amyotrophic lateral sclerosis, Heart rate variability, Autonomic function, Sympathetic hyperactivity, Sympathovagal imbalance

## Abstract

**Background:**

Patients with amyotrophic lateral sclerosis (ALS) occasionally exhibit autonomic nervous system dysregulation. We examined whether autonomic regulation differed across patients with ALS with varying severity and progression.

**Methods:**

A total of 45 patients with ALS were enrolled and classified into three subgroups using cluster analysis. Heart rate variability was assessed using the maximum entropy method. The low-frequency (LF) and high-frequency (HF) components, LF/HF ratio (LF/HF), and heart rate (HR) were measured. Temporal changes in each parameter during rest, mental tasks, and post-task rest were evaluated. The values for all patients and subgroups were compared with those of 11 healthy control subjects. Between-group differences were evaluated at rest and using the Task/Rest and After/Task ratios, and within-group changes across the three phases were also analyzed, with non-parametric statistical tests applied.

**Results:**

Cluster analysis classified the patients into three groups: “Group 1: early-preserved group”, “Group 2: late-preserved group”, and “Group 3: late-impaired group”. Overall, the patients showed lower HF and higher LF/HF at rest than the controls, indicating parasympathetic hypoactivity and sympathetic predominance. Abnormalities were more prominent in Groups 1 and 3 than in Group 2. The former two groups showed blunted HF, LF/HF and HR responses during the tasks. The late-preserved group (Group 2) showed no difference in the Task/Rest ratios of HF, LF/HF and HR compared with the controls.

**Conclusion:**

Autonomic regulatory functions differ depending on the severity and progression of ALS. The presence of HRV abnormalities in early-preserved patients suggests that autonomic dysregulation in ALS may not be limited to a late-stage secondary complication but may also be present earlier stages. Recognizing HRV abnormalities from early stages may help identify patients at risk of faster progression. Future longitudinal studies in larger cohorts are needed to establish the pathophysiological significance of HRV abnormalities.

**Supplementary Information:**

The online version contains supplementary material available at 10.1186/s12883-026-04901-w.

## Introduction

Amyotrophic lateral sclerosis (ALS) is a progressive neurodegenerative disorder affecting motor neurons. However, recent studies have indicated that it may be involved in multisystem degeneration affecting autonomic and sensory neurons [1, 2]. Non-motor symptoms unrelated to motor neuron impairment are observed in 5%–80% of patients with ALS [3] and significantly reduce their quality of life [4]. Autonomic symptoms of ALS include cardiovascular, gastrointestinal, voiding, and sudomotor dysfunction [5]. Urinary and gastrointestinal symptoms are noted in 30% of patients with ALS [6]. While these autonomic dysfunctions may appear mild, some patients with tracheostomy and invasive ventilation (TIV) in the advanced stages may present prominent fluctuations of blood pressure and heart rate, known as “autonomic storm,” which can lead to sudden death [7]. This condition is caused by central sympathetic hyperactivity and downregulation of peripheral sympathetic receptor function, which may result in circulatory collapse [8, 9]. Moreover, since unstable blood pressure is associated with disease progression [10], autonomic dysfunction is a significant concern in ALS.

Recent studies have reported an imbalance between the sympathetic and parasympathetic nervous functions in ALS [5]. Assessment of heart rate variability (HRV), a non-invasive measure of autonomic function, revealed that patients with ALS exhibited reduced autonomic activity with sympathetic predominance compared to healthy individuals [11–14]. Neurophysiological studies indicated that muscle sympathetic nerve activity (MSNA) and skin sympathetic nerve activity (SSNA) initially increased and then decreased with age and disease duration [15, 16]. Secondary factors such as psychological stress, long-term ventilatory support, severe muscle atrophy, long-term bedridden state, and repetitive infections may contribute to sympathetic hyperactivity [10, 17]. Although these autonomic abnormalities are evident, including sympathetic hyperactivity and concomitant/subsequent blunted vascular responses, the mechanisms and factors underlying these autonomic dysfunctions remain unclear.

Focusing on patients with ALS at the stages before ventilator use, previous studies reported that decreased HRV was associated with decreased lung capacity and increased disease duration [18, 19]. In contrast, a study found respiratory dysfunction was not associated with decreased parasympathetic activity [20]. Considering the heterogeneous progression of ALS, several additional measures that capture ALS progression must be considered. Although most studies examined autonomic function at rest, evaluating autonomic function in response to tasks may provide a more comprehensive assessment. Understanding the factors associated with autonomic dysregulation may help clarify the clinical significance and fundamental pathomechanism of the autonomic dysfunction in ALS.

This study examined whether autonomic dysregulation differs across patients with ALS with varying severity and progression using non-invasive HRV measurements. This study aimed to identify factors associated with autonomic dysregulation in ALS by assessing sympathetic and parasympathetic nervous system functions under task conditions.

## Methods

### Participants

We enrolled 45 patients who visited the Tokyo Metropolitan Neurological Hospital between March 2017 and September 2025 and were diagnosed with sporadic ALS. Although the enrollment of patients was not consecutive, we enrolled patients who provided written informed consent to participate in the study. All patients were diagnosed with ALS according to the revised El Escorial criteria as “clinically definite,” “clinically probable,” “clinically probable-laboratory supported,” or “clinically possible” ALS [21]. Patients with a history of alcohol or tobacco abuse, arrhythmias, or peripheral neuropathy were excluded. There were no patients who were taking medications that could affect autonomic functions at the time of investigation. All the participants maintained sufficient cognitive function to meet the task requirements.

The following clinical characteristics were assessed: sex, age at onset, age at evaluation, height, premorbid weight, body weight at diagnosis, body weight at evaluation, disease duration (months), onset region (bulbar, upper or lower limb), use of enteral nutrition (EN), use of non-invasive ventilation (NIV), the Revised Amyotrophic Lateral Sclerosis Functional Rating Scale (ALSFRS-R) at evaluation [22], and seated forced vital capacity (FVC) at evaluation. Thereafter, body mass index (BMI) was calculated as weight (kg)/height (m)^2^. FVC was expressed as a percentage and calculated as follows: (measured FVC/predicted FVC) × 100. ΔALSFRS-R was calculated as follows: (48 – ALSFRS-R score at evaluation)/disease duration (months). ΔFVC was calculated as (100 – measured FVC)/disease duration (months) and ΔBMI as (premorbid BMI – BMI at evaluation)/disease duration (months).

Eleven healthy subjects (median 59.0 (IQR 10.5) years, eight males) served as the control population [23]. This study was approved by the ethics committee of the Tokyo Metropolitan Neurological Hospital (No. R03-019) and the Tokyo Metropolitan Institute of Medical Science (No. 22-19). This study was performed according to the ethical standards described in the latest version of the Declaration of Helsinki and the Ethical Guidelines for Clinical Research of the Tokyo Metropolitan Neurological Hospital. All participants provided written informed consent before participating in the study.

### Heart rate variability measurement

HRV measurements were conducted in the outpatient examination room between 13:00 and 17:00 after routine consultations. Patients lay on a bed, and a quiet environment was set up for HRV measurements in a relaxed state. The three-behavioral-state paradigm consisting of the ‘Rest,’ ‘Task,’ and ‘After’ phases was applied based on previous studies [23–25]. During the Rest phase, patients relaxed on a bed for 120 s. For the Task phase, a 60-second silent Serial Sevens Test was performed in patients’ minds at their own pace. Finally, during the After phase, patients were instructed to cease the calculation and relax again for 120 s. The Serial Sevens Test, a mental arithmetic task with a high cognitive load [26], was used to assess mental stress in patients [27]. In the present study, each patient performed calculations in mind from ‘100 minus 7,’ ‘93 minus 7,’ and so on. We verified task completion by asking the patient the final number after the measurement was completed. Control subjects performed a random number generation task during the Task phase, as previously reported [24, 25]. Data on control subjects were obtained from previous independent studies conducted by one of the co-authors [23]. Although the tasks’ characteristics differed between patients and control subjects in this study, we judged that the two tasks had equivalent loads to mental tasks.

Spectral analysis of the HRV, which is widely used to assess autonomic function, was conducted using an ECG monitor (RF-ECG2; GM3, Tokyo, Japan) attached to the chest. ECG data were recorded on a computer, and R-R intervals were analyzed using the maximum entropy method (MemCalc, GMS, Tokyo, Japan) [28]. This method allows the analysis of short, 30-second data segments [29] and was applied to the three-behavioral-state paradigm in this study [24, 25]. MemCalc calculated the low-frequency (LF) and high-frequency (HF) components every two seconds by integrating power within frequency ranges of 0.04–0.15 Hz for LF and 0.15–0.4 Hz for HF. The heart rate (HR, bpm) was calculated from the R-R intervals [28].

### Statistical analysis

We utilized Uniform Manifold Approximation and Projection (UMAP) and Ordering Points To Identify the Clustering Structure (OPTICS) algorithms to characterize the clinical state of patients with ALS [30]. Using UMAP, nine variables that reflect the severity and progression of ALS (age, disease duration, ALSFRS-R, FVC, BMI, ΔBMI, and three binary variables: bulbar onset, EN use, and NIV use) were reduced to a two-dimensional space, allowing for visualization of the data structure [29]. In the dataset used, the FVC values were missing for two people (5.7% of the total); therefore, we used the k-nearest neighbor (KNN) method to fill in the missing values [31, 32]. A composite distance metric combining the Euclidean distance for continuous variables and the Hamming distance for binary variables was applied, with the five nearest neighbors specified for each data point and a minimum distance parameter of 0.01. OPTICS was then applied to the two-dimensional UMAP embedding, with the minimum number of points required to form a cluster (minPts) set to 5% of the total sample size and the maximum neighborhood radius (eps) set to 5. OPTICS analyzes the density structure of the data and generates a reachability plot. Clustering structures were visualized using this plot, and clusters were extracted by applying Density-Based Spatial Clustering of Applications with Noise (DBSCAN) as a cluster extraction method to the OPTICS ordering, using a reachability distance threshold (ε_cl) of 2.5 [33].

Given the exploratory and descriptive nature of this study, we examined correlations between autonomic function measures and clinical variables in the overall ALS patients. Spearman correlation analyses were performed to assess associations of HRV and HR values (measured at rest, during the task, and after the task) with clinical variables, including clustering variables (age, disease duration, ALSFRS-R, FVC, BMI, ΔBMI, bulbar onset, enteral nutrition [EN] use, and noninvasive ventilation [NIV] use) and additional indices of disease progression (ΔALSFRS-R and ΔFVC). Next, univariate and multivariate linear regression analyses were performed for disease-progression parameters (∆ALSFRS-R, ∆FVC, and ∆BMI) using HRV and HR values as independent variables in the overall ALS patients to confirm the relationships between autonomic function measures and clinical variables for disease progression rate.

After assigning the patients to clusters, nine variables were summarized for each cluster. Differences in distributions between clusters were assessed to characterize the disease states. The Shapiro-Wilk test was used to evaluate variable distributions, guiding the choice of statistical methods. The Kruskal-Wallis test was used to compare continuous data between clusters, whereas the chi-squared test was used to analyze categorical data. In addition, age and sex were compared between control subjects and the overall ALS patients using the Mann–Whitney U test and chi-squared test, respectively.

We compared the resting HRV and resting HR values of the following groups to evaluate the differences in autonomic nervous dysfunction between patients with ALS and control subjects: control subjects vs. all patients and control subjects vs. each cluster. The Mann–Whitney U test was used for comparison. Next, we compared the changes in HRV and HR values in the three phases (Rest, Task, and After) between control subjects, all patients with ALS, and each cluster. We used Friedman’s repeated-measures test for this analysis and Nemenyi’s test for post-hoc comparisons.

Additionally, we compared the changes in HRV and HR values using the ratio of values in the Task phase to the Rest phase (Task/Rest) and the After phase to the Task phase (After/Task). Comparisons between control subjects and all patients were performed using the Mann-Whitney U test. In contrast, comparisons between control subjects and each cluster were performed using the Kruskal–Wallis test and a post-hoc Dunn’s test.

As supplementary analyses, we conducted two complementary approaches to evaluate between-cluster differences in task-related responses. First, we performed a regression-based analysis using participant-level phase-to-phase ratios (Task/Rest and After/Task) for each HRV and HR value and compared these ratios between clusters using adjusted linear regression models (Model 1: age and sex; Model 2: additionally adjusted for BMI and baseline HR). Second, we fitted linear mixed-effects models with phase, cluster, and their interaction, adjusting for age, sex, BMI, and disease duration and including a participant-level random intercept. Using estimated marginal means, we compared phase-to-phase change scores (Task/Rest; After/Task) between clusters (Kenward–Roger df, 95% CIs).

All statistical analyses were conducted using R software (version 4.4.0) with the Uwot and DBSCAN packages [34], with the significance level set at *p* < 0.05.

## Results

### Patient characteristics

The clinical characteristics of the patients are summarized in Table 1. The median age at examination was 64.0 (IQR 15.0) years. While eleven patients were using NIV, all used it either only at night or intermittently during the day, and none were using it at the time of the examination. Among the 19 patients using EN, 18 used gastrostomy tubes, and one used a nasogastric tube; all patients were in stable condition without pain or wound inflammation following gastrostomy tube placement. There was no significant difference in sex distribution between the control group and the overall ALS cohort (*p* = 0.340), whereas the ALS cohort was older than the control group (*p* = 0.023).

**Table 1:** Patient characteristics

	Overall ALS patients (n = 45)		Cluster groups						*p* value
			Group 1 Early-preserved (n = 18)		Group 2 Late-preserved (n = 10)		Group 3 Late-impaired (n = 17)		
Male	23	(51.1)	11	(61.1)	4	(40.0)	8	(47.1)	0.515
Age at onset (years)	62.0	(12.0)	62.0	(15.5)	55.5	(16.8)	63.0	(6.0)	0.484
Age at examination (years)	64.0	(15.0)	62.0	(14.8)	59.5	(16.8)	66.0	(9.0)	0.758
Disease duration (months)	27.0	(28.0)	15.0	(10.3)	51.5	(14.5)	45.0	(23.0)	<0.001
ALSFRS-R	38.0	(12.0)	40.5	(7.3)	38.0	(2.5)	23.0	(9.0)	<0.001
ΔALSFRS-R	0.4	(0.5)	0.4	(0.5)	0.2	(0.1)	0.5	(0.4)	<0.001
BMI (kg/m^2^)	20.4	(4.5)	21.6	(4.4)	22.2	(3.5)	19.5	(2.6)	0.007
ΔBMI (kg/m^2^)	0.04	(0.10)	0.03	(0.20)	0.01	(0.03)	0.07	(0.06)	0.034
FVC (%)	77.5	(40.1)	87.8	(21.6)	100.7	(14.0)	39.5	(15.1)	<0.001
ΔFVC (%)	0.95	(1.4)	1.00	(1.8)	-0.01	(0.3)	1.20	(1.4)	<0.001
EN	19	(42.2)	2	(11.1)	2	(10.0)	15	(88.2)	<0.001
NIV	11	(24.4)	2	(11.1)	0	(0.0)	9	(52.9)	0.001
Onset site									
Upper limb	22	(48.9)	11	(61.1)	6	(60.0)	5	(29.4)	0.125
Lower limb	12	(26.7)	4	(22.2)	4	(40.0)	4	(25.5)	0.555
Bulbar	13	(28.9)	5	(27.8)	1	(10.0)	7	(41.2)	0.223

Data are represented as median (interquartile rnage) or number (percentage). Group comparisons were performed using the Kruskal-Wallis test for non-parametric data or Chi-square test as appropriate

*ALS* Amyotrophic lateral sclerosis, *ALSFRS-R* Amyotrophic Lateral Sclerosis Functional Rating Scale-Revised, *ΔALSFRS-R* monthly ALSFRS-R decline, *BMI* Body-mass index, *ΔBMI* monthly  BMI decline, *FVC* predicted forced vital capacity, *ΔFVC* monthly FVC  decline, *EN* Enteral nutrition, *NIV* Noninvasive ventilation

**Fig. 1:**
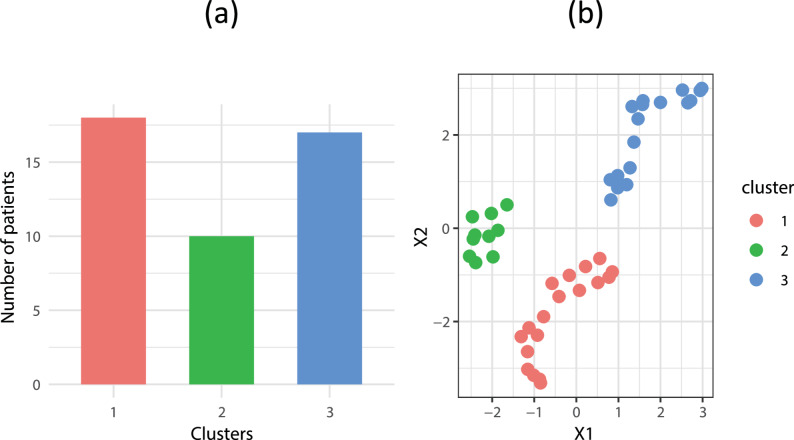
**a** Distribution of clusters detected by Ordering Points To Identify the Clustering Structure (OPTICS) on the two-dimensional reduced representation of the study data. **b** Uniform Manifold Approximation and Projection clusters for the two-dimensional reduced representation of the data annotated by the clusters generated by OPTICS. X1 and X2 denote the two dimensions of the UMAP embedding used for visualization; their scales do not correspond to specific clinical variables

We identified three subgroups (clusters) among the patients using the OPTICS clustering algorithm. The distribution of clusters is shown in Fig. 1(a). In Fig. 1(b), the clusters detected by OPTICS are visualized by performing UMAP on a two-dimensional reduced data representation. Patient profiles for each cluster are shown in Table 1. The disease duration, ALSFRS-R, ΔALSFRS-R, BMI, ∆BMI, FVC, ΔFVC, the number of patients using EN and NIV significantly differed between the clusters. Cluster 1 exhibited the shortest disease duration (15.0 (10.3) months), with relatively preserved motor neuron function, as indicated by ALSFRS-R score (40.5 (7.3)), BMI (21.6 (4.4) kg/m2), and FVC (87.8% (21.6)). In contrast, clusters 2 and 3 had longer disease durations (51.5 (14.5) months and 45.0 (23.0) months, respectively). Cluster 2 showed preserved ALSFRS-R scores 38.0 (2.5), BMI (22.2 (3.5) kg/m2), and FVC (100.7% (14.0)). Cluster 3 demonstrated advanced symptoms with a low ALSFRS-R score (23.0 (9.0)), low BMI (19.5 (2.6) kg/m2), and low FVC (39.5% (15.1)). Based on these characteristics, three groups were named as follows: “Group 1: early-preserved group” (*n* = 18), “Group 2: late-preserved group” (*n* = 10), and “Group 3: late-impaired group” (*n* = 17).

### Heart rate variability measurement

Table 2 shows the HRV and HR at the Rest phase in the control subjects and patients. The overall ALS patients showed lower HF and higher LF/HF, regardless of normal LF, compared with controls. The results of Spearman correlation analyses between clinical variables and autonomic function measures in the overall ALS patients are shown in Supplementary Table 1. HF showed modest but significant correlations with ∆ALSFRS-R, ∆BMI, and ∆FVC, which are indicators of disease progression, across all three phases. LF/HF and HR were also correlated with ∆BMI and ∆FVC across all three phases, except for the association between LF/HF and ∆BMI during the Task phase. Univariate linear regression analyses in the overall ALS patients demonstrated significant associations of HF with ∆ALSFRS-R and ∆FVC, and of LF/HF with ∆FVC and ∆BMI (Supplementary Table 2). Multivariate linear regression analyses in the overall ALS patients, with disease progression parameters as dependent variables and sex, age, HF, LF/HF, and HR as independent variables, showed no significant associations between HRV measures and disease progression parameters (Supplementary Table 3).

**Table 2:** Group comparisons of heart rate variability and heart rate at Rest phase

	Control subjects (n = 11)		Overall ALS patients (n = 45)			Group 1 Early-preserved(n = 18)			Group 2 Late-preserved (n = 10)			Group 3 Late-impaired (n = 17)		
					*p* value			*p* value			*p* value			*p* value
HF	135.00	(102.0)	47.99	(138.2)	0.008	45.90	(54.3)	0.003	163.60	(237.3)	0.916	14.45	(87.1)	0.006
LF	129.28	(136.3)	117.20	(197.1)	0.302	124.71	(163.7)	0.458	225.31	(175.6)	0.647	70.66	(85.7)	0.060
LF/HF	1.28	(1.1)	3.18	(3.6)	0.002	3.47	(3.2)	0.004	1.80	(0.7)	0.170	3.73	(4.2)	0.001
HR	72.70	(10.7)	76.89	(17.7)	0.201	76.78	(19.0)	0.216	70.56	(9.5)	0.597	82.04	(27.3)	0.048

Data are represented as median values (interquartile range)

Group comparisons were performed between the control group and each ALS group using the Mann-Whitney U test

*ALS* Amyotrophic lateral sclerosis, *HF* High-frequency component, *LF* Low-frequency component, *HR* Heart rate

At the subgroup level, pronounced abnormalities were observed in Groups 1 and 3. Both groups had lower HF (*p* = 0.003 and *p* = 0.006, respectively) and higher LF/HF (*p* = 0.004 and *p* = 0.01, respectively) than controls. LF in Group 3 was low but not significantly different from the control value. In contrast, Group 2 showed no significant differences in any parameter with the controls. The HR in the Rest phase showed no significant differences in Groups 1 and 2, but a significant difference in Group 3 compared with controls (*p* = 0.048).

**Fig. 2:**
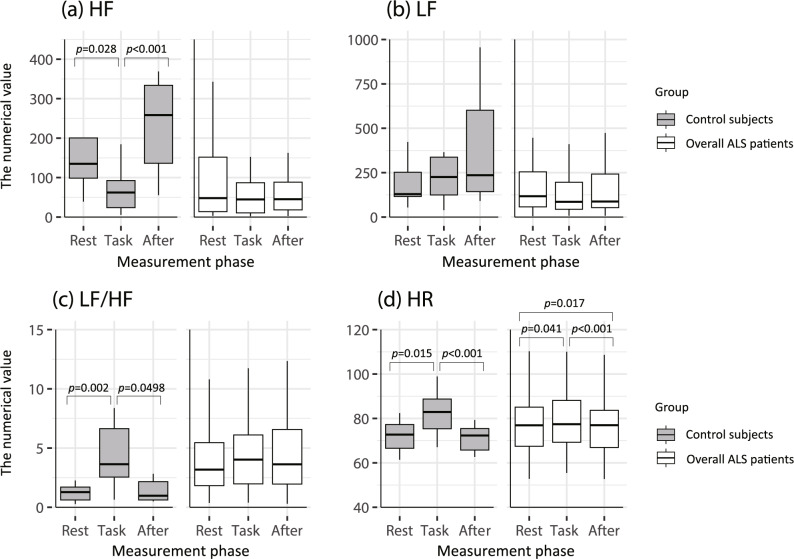
Comparisons of heart rate variability (HRV) across the Rest, Task, and After phases in control subjects and overall ALS patients with amyotrophic lateral sclerosis. High-frequency (HF, **a**), low-frequency (LF, **b**), LF/HF ratio (**c**), and heart rate (HR, **d**) were compared across three-time points (Rest, Task, and After) for each group. Statistical analysis was conducted using the Friedman test to assess within-cluster temporal changes

Figure 2 shows the variations in HRV and HR at the Rest, Task, and After phases in the control subjects and overall patients with ALS. In control subjects, HF significantly decreased from Rest to Task (Fig. 2a, *p* = 0.028) and increased from Task to After (Fig. 2a, *p* < 0.001). In contrast, LF/HF increased from Rest to Task (Fig. 2c, *p* = 0.002) and decreased from Task to After (Fig. 2c, *p* = 0.0498), although LF showed no significant changes between each phase (Fig. 2b). HR increased in the Task phase and returned to the baseline value in the After phase (Fig. 2d). In all patients with ALS, no variations across the phases were observed for HF, LF, and LF/HF (Fig. 2a-c). There were significant variations only in the HR in the patients (Fig. 2d). Table 3 compares the ratio of each value at Task/Rest and After/Task. Significant differences in the Task/Rest and After/Task ratios were observed between control subjects and the overall ALS patients in HF (*p* < 0.001 and *p* < 0.001, respectively), LF/HF (*p* < 0.001 and *p* = 0.002, respectively), and HR (*p* < 0.001 and *p* < 0.001, respectively).

**Fig. 3:**
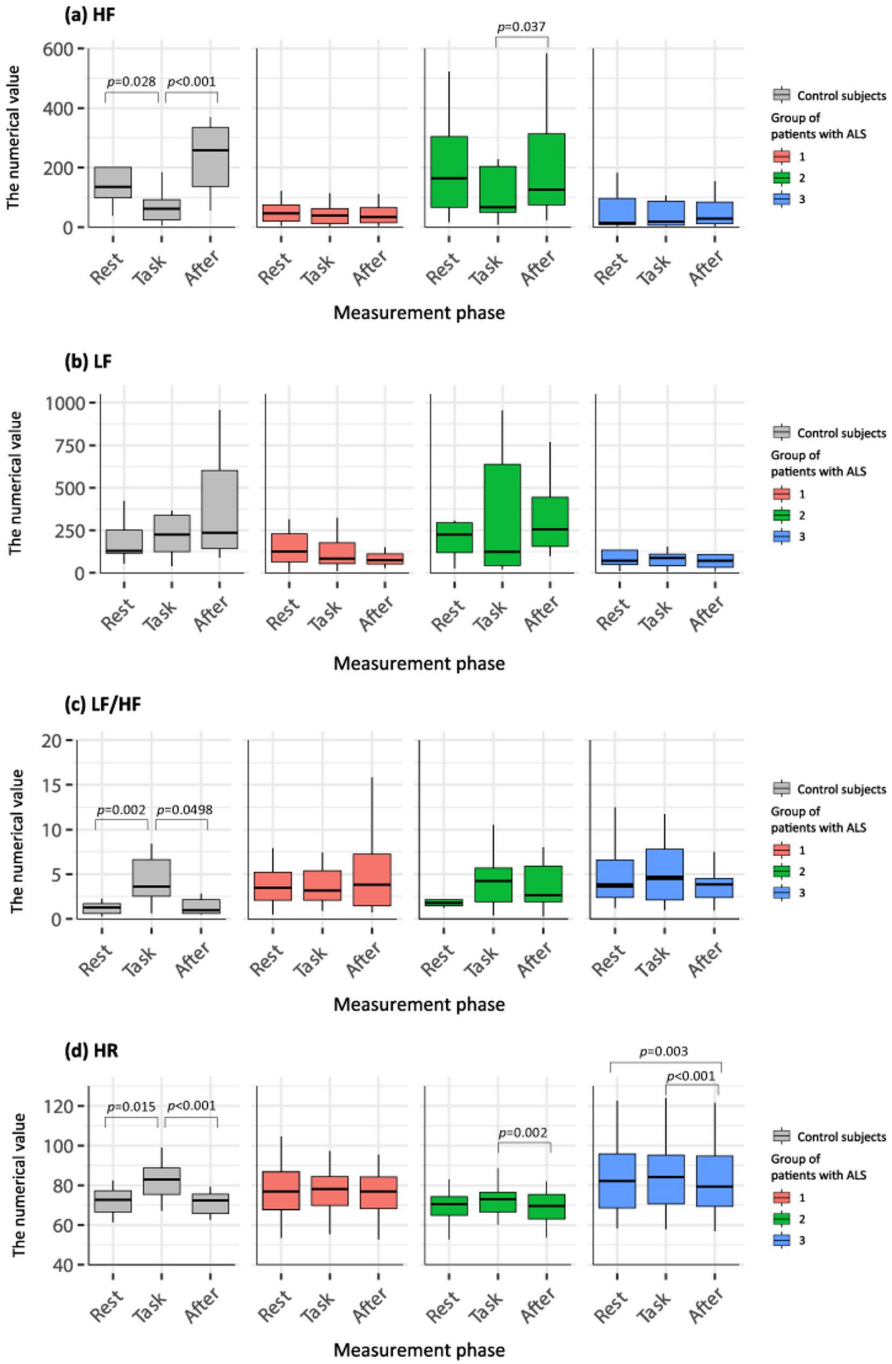
Comparisons of heart rate variability (HRV) across the Rest, Task, and After phases in control subjects and each group. High-frequency (HF, **a**), low-frequency (LF, **b**), LF/HF ratio(**c**), and heart rate (HR, **d**) were compared across three-time points (Rest, Task, and After) for each group. Statistical analysis was conducted using the Friedman test to assess within-cluster temporal changes

Figure 3 shows the variations in HRV and HR in each patient group. Among the three groups, only Group 2 showed a significant increase in HF at the After phase, similarly to the control subjects (Fig. 3a, *p* = 0.037). For LF and LF/HF, no significant changes were observed in any groups (Fig. 3b, c), although in Group 2, the LF/HF at the Task phase was non-significantly higher than at the Rest phase (Fig. 3c). As shown in Fig. 3d, the variations in HR across the phases in each group were minimal compared to those in the control subjects. Table 3 compares the ratio of values at Task/Rest and After/Task in the control group and each patient group. Significant differences in the Task/Rest and After/Task ratios were observed between groups for HF (*p* = 0.005 and *p* < 0.001, respectively), LF/HF (*p* = 0.004 and *p* = 0.020, respectively), and HR (*p* < 0.001 and *p* < 0.001, respectively). Furthermore, the post hoc test using Dunn’s test revealed significant differences in all variables between the control subjects and Group 1 and between the control subjects and Group 3. Between-group comparisons of phase-to-phase ratios based on adjusted linear regression models are shown in Supplementary Table 4. These analyses indicated cluster-specific differences in several ratio outcomes. In particular, compared with Group 1, Group 2 showed higher ratios for HR and LF/HF at Task/Rest and higher ratios for LF at After/Task, while showing lower ratios for HR at After/Task. These findings were broadly consistent after additional adjustment for BMI and baseline HR, although the HR (Task/Rest) contrast was attenuated and no longer statistically significant in Model 2.

**Table 3:** Group comparisons of ratios of heart rate variability and heart rate between control subjects and patients with ALS

	Control subjects(n = 11)		Overall ALS patients (n = 45)		*p* value*	Group 1 Early-preserved (n = 18)		Group 2 Late-preserved (n = 10)		Group 3 Late-impaired (n = 17)		*p* value**	Post-hoc test (*p* value only)***		
													Control vsGroup 1	ControlvsGroup 2	Control vsGroup 3
HF															
Task/Rest	0.26	(0.26)	0.87	(0.62)	<0.001	0.87	(0.69)	0.73	(0.50)	1.01	(0.49)	0.005	0.010	0.120	0.002
After/Task	4.09	(3.70)	1.07	(0.79)	<0.001	0.98	(0.85)	1.37	(0.41)	0.93	(1.01)	<0.001	<0.001	0.032	<0.001
LF															
Task/Rest	0.85	(1.97)	0.91	(0.98)	0.695	0.91	(0.87)	1.19	(1.74)	0.99	(0.93)	0.911	-	-	-
After/Task	1.15	(2.30)	0.94	(0.84)	0.342	0.97	(0.80)	1.6	(2.77)	0.87	(0.62)	0.220	-	-	-
LF/HF															
Task/Rest	4.87	(3.58)	1.19	(1.08)	<0.001	1.06	(0.78)	1.87	(2.95)	1.06	(0.62)	0.004	0.005	0.221	0.002
After/Task	0.25	(0.53)	0.88	(1.18)	0.002	0.9	(1.25)	1.3	(2.27)	0.79	(0.64)	0.020	0.013	0.050	0.032
HR															
Task/Rest	1.13	(0.09)	1.01	(0.04)	<0.001	1.01	(0.04)	1.03	(0.05)	1.01	(0.03)	<0.001	<0.001	0.160	<0.001
After/Task	0.88	(0.10)	0.98	(0.04)	<0.001	0.98	(0.05)	0.94	(0.06)	0.98	(0.02)	<0.001	<0.001	0.423	<0.001

Data are represented as median values (interquartile range)

*ALS* Amyotrophic lateral sclerosis, *HF* High-frequency component, *LF* Low-frequency component, *HR* heart rate

*Group comparisons were performed between the control group and overall ALS patients using the Mann-Whitney U test. **Group comparisons were performed using the Kruskal-Wallis test. ***Post-hoc test were performed using the Dunn test for pair of groups that had a significant on Kruskal wallis tets

Results of the exploratory adjusted linear mixed-effects models examining phase-to-phase changes are provided in Supplementary Table 5. These analyses indicated that Group 2 showed a larger Task/Rest change in LF than Group 1, with between-group differences in HR for both Task/Rest and After/Task changes. No statistically significant between-group differences were observed for HF or LF/HF.

## Discussion

This study investigated task-related autonomic reactivity in patients with ALS and examined whether HRV and HR responses differ across clinically defined clusters. Overall, patients with ALS had lower HF and higher LF/HF ratios than the controls. LF reflects sympathetic and parasympathetic activity, whereas HF reflects parasympathetic activity [35]. It has been suggested that patients with ALS show a shift in autonomic balance toward sympathetic hyperactivity and parasympathetic hypoactivity, or sympathovagal imbalance [11–14]. The blunted response of LF/HF response during the task in patients might have been caused by ceiling effects due to high LF/HF [27]. Our findings suggest that autonomic functions may differ according to the severity and progression of ALS. The abnormalities tended to be more pronounced in Group 1 (early-preserved group) and Group 3 (late-impaired group) than in Group 2 (late-preserved group). Alterations in autonomic function may be related to the variability in ALS disease progression. However, as the clustering approach is exploratory, the findings should be interpreted with caution.

Accumulating evidence from previous neurophysiological studies has established that patients with ALS exhibit sympathetic hyperactivity and sympathovagal imbalance. Our findings show that the decreased HF and increased LF/HF ratios are consistent with previous reports [13]. Although the pathophysiology of these autonomic dysregulations has not been clarified, limbic system abnormalities have been suggested as a probable etiology of sympathetic hyperactivity [7, 10]. ALS is a multisystem disorder involving frontotemporal lobes and central sensory and autonomic pathways [1, 7]. The brain centers of autonomic regulation, including the insular cortex, cingulate gyrus, hypothalamus, central grey matter, and brainstem autonomic centers, may be involved, at least functionally and even pathologically, in the advanced stages of ALS [36].

The results at the Rest phase showed abnormal findings in Groups 1 and 3, but not in Group 2. The abnormalities in the overall ALS patients might reflect the results in Groups 1 and 3. While having a short disease duration, Group 1 (the early-preserved group) showed large values of ∆ALSFRS-R, ∆BMI, and ∆FVC, comparable to those in the late-impaired group. This finding indicates that this group may include patients with relatively rapid disease progression, in whom alterations in autonomic regulatory functions were observed even at early stages of ALS. Psychological stress and the disease pathophysiology might contribute to sympathetic hyperactivity [7, 17]. Group 3 (the late-impaired group) included typical advanced cases of ALS with long disease duration, lower ALSFRS-R, lower BMI, and lower FVC. This group showed markedly lower HF and higher LF/HF at rest and blunted LF/HF and HR responses than controls, similarly to the results in Group 1. This similarity indicates that Group 1 patients may develop clinical characteristics similar to those in Group 3 along with disease progression. Although clinical variables such as ALSFRS-R and FVC were included in the clustering process, the presence of autonomic dysfunction in Group 1 despite relatively preserved motor and respiratory function suggests that autonomic impairment may not simply reflect disease severity but may represent a partially independent pathological process in ALS. Exploratory correlation analyses further showed the associations between HRV indices and disease progression-related variables. Univariate linear regression also demonstrated significant associations between HF and ∆ALSFRS-R and ∆FVC, and between LF/HF and ∆FVC and ∆BMI, although multivariate analyses failed to show significant associations (*p* < 0.1). These results suggest that autonomic functional measures may serve as surrogate markers of disease progression, although their role as predictive biomarkers for ALS requires validation in larger prospective cohorts.

Group 2, the late-preserved group, showed different results from the other groups. None of the HRV values at rest in Group 2 showed significant differences from the control values. Furthermore, HF showed a significant increase in response to the end of the task load, and the ratio of values at Task/Rest and After/Task showed smaller differences from the controls compared to Groups 1 and 3. These results suggest that this group exhibited autonomic regulation similar to healthy subjects. Despite the long disease duration, this group maintained better motor and respiratory function than the others. Previous studies reported that patients with ALS and FVC < 50% showed lower HRV compared to those with FVC ≥ 50% [18]. Considering that HRV is affected by respiratory dysfunction, the preserved respiratory function in Group 2 may have contributed to ameliorated autonomic dysfunction. Supplementary Tables 4 and 5 present additional analyses conducted to complement the findings in Fig. 3 by testing between-cluster contrasts in task-related response ratios and response patterns. Although some contrasts were attenuated after adjustment, these analyses suggest that task-related reactivity may differ across clusters. In addition, part of the observed between-cluster differences may be related to underlying clinical characteristics or pathway-related factors, such as body mass index and baseline heart rate.

Group 1, the early-preserved group, demonstrated autonomic abnormalities despite relatively preserved motor function, suggesting that dysregulation can emerge at early disease stages. Given their rapid progression, these patients may eventually resemble Group 3, the late-impaired group. In clinical practice, disease-modifying therapies might slow ALS progression, potentially enabling a shift toward the late-preserved phenotype and maintaining autonomic stability. Although parasympathetic dysfunction is known to worsen with disease progression [20], autonomic regulation in our cohort showed variability, with some patients exhibiting compromised function even at early stages and others maintaining it despite long disease duration. Further longitudinal studies are required to clarify whether early therapeutic interventions can alter HRV abnormalities and improve prognosis.

This study had some limitations. First, the sample size was small. Although our sample may not strictly represent the entire ALS population, the use of cluster analysis allowed for the consideration of heterogeneity within the patient group, which is a strength of this study. However, the clustering approach was exploratory in nature, and the variables used to define the clusters were also examined to describe their clinical characteristics rather than to provide independent validation of cluster robustness. Therefore, the potential for selection bias, limited external validity, and lack of independent validation should be acknowledged, and further validation using larger, independent cohorts is warranted. Second, the task differed between controls and patients. Control subjects performed a random number generation task, whereas patients performed a serial seven test. In this study, we considered both tasks equivalent to mental loads. In addition, although task completion was confirmed after testing, objective measures of cognitive engagement or task performance were not assessed. Therefore, the effective cognitive load of the mental task may have varied across individuals due to differences in attention, concentration, or cognitive function, which could have influenced HRV responses. Third, we did not investigate the psychological symptoms of patients. Psychological stress, anxiety, and irritation may influence HRV [23], highlighting the need to incorporate such measures into future investigations. Fourth, the age of the controls was lower than that of patients with ALS. Aging has been reported to affect autonomic function. The analyses in this study were conducted without adjusting for age differences, which may have influenced the results.

This study suggests that autonomic regulatory functions may differ depending on the varying severity and progression of ALS. Patients with preserved overall function and BMI, even those with long disease duration, may be less susceptible to autonomic dysfunction. However, the clustering results in this study are exploratory in nature and are not intended to inform clinical decision-making at this stage. Longitudinal studies are needed to elucidate this possibility.

## Supplementary Information

Supplementary Material 1.

Supplementary Material 2.

Supplementary Material 3.

Supplementary Material 4.

Supplementary Material 5.

## Data Availability

Data supporting the findings of this study are available upon request from the corresponding authors.The data are not publicly available because they contain information that can compromise the privacy of the research participants.

## Abbreviations

ALS: Amyotrophic lateral sclerosis
ALSFRS-R: The Revised Amyotrophic Lateral Sclerosis Functional Rating Scale
BMI: Body mass index
EN: Enteral nutrition
FVC: Forced vital capacity
HF: High-frequency
HR: Heart rate
HRV: Heart rate variability
KNN: K-nearest neighbor
LF: Low-frequency
LF/HF: LF/HF ratio
MSNA: Muscle sympathetic nerve activity
NIV: Non-invasive ventilation
OPTICS: Ordering Points To Identify the Clustering Structure
SSNA: Skin sympathetic nerve activity
TIV: Tracheostomy and invasive ventilation
UMAP: Uniform Manifold Approximation and Projection
